# Evaluation of Mode II Fracture Toughness of Hybrid Fibrous Geopolymer Composites

**DOI:** 10.3390/ma14020349

**Published:** 2021-01-12

**Authors:** Sallal R. Abid, Gunasekaran Murali, Mugahed Amran, Nikolai Vatin, Roman Fediuk, Maria Karelina

**Affiliations:** 1 Civil Engineering Department, Wasit University, Kut 52001, Iraq; sallal@uowasit.edu.iq; 2 School of Civil Engineering, SASTRA Deemed University, Thanjavur 613401, India; 3 Department of Civil Engineering, College of Engineering, Prince Sattam Bin Abdulaziz University, Alkharj 11942, Saudi Arabia; m.amran@psau.edu.sa; 4 Department of Civil Engineering, Faculty of Engineering and IT, Amran University, Quhal 9677, Amran, Yemen; 5 Higher School of Industrial, Civil and Road Construction, Peter the Great St. Petersburg Polytechnic University, 195251 St. Petersburg, Russia; vatin@mail.ru; 6 Polytechnic Institute, Far Eastern Federal University, 690922 Vladivostok, Russia; roman44@yandex.ru; 7 Department of Machinery Parts and Theory of Mechanisms, Moscow Automobile and Road Construction University, 125319 Moscow, Russia; Karelinamu@mail.ru

**Keywords:** fracture toughness, geopolymer concrete, glass fiber, polypropylene fiber, steel fiber

## Abstract

This research aims to examine the fracture toughness of hybrid fibrous geopolymer composites under mode II. For this purpose, eight geopolymer mixtures were cast and tested to evaluate the influence of steel and synthetic fiber hybridization on mode II fracture response. The first mixture was plain and was kept as a reference, while steel, polypropylene and glass fibers were used in the rest seven mixtures. The first three of which were mono-reinforced with one of the three fibers, while the rest of the four were hybrids reinforced with combinations of steel and synthetic fibers. The Brazilian center notched disc and the double notched cube test configurations were used to evaluate the mode II fracture toughness of the eight mixtures. The results of the tests showed that steel fibers played the vital role in enhancing the fracture toughness, where the mixtures S1.6 and S1.3G0.3 showed the best performance. The results also showed that increasing the notch depth decreased the fracture toughness with an approximate linear decrement fashion. It was found that the use of double-notched cubes resulted in much higher fracture toughness than the Brazilian notched discs, where the ratio of normalized fracture toughness of the disc specimens to cube specimens was approximately 0.37 to 0.47. This is attributed to the concentration of stresses along one defined path in the disc specimens compared to the multi-path stresses in the cube specimens. In addition, the accompanied tensile stresses in the disc specimens may lead to a mode I fracture before the designed mode II fracture.

## 1. Introduction

Growth in the population and the new international regulations of recent construction require efficient and energy-saving materials. An increasing worldwide demand for cement production will lead to a need for 6.1 billion metric tons of cement in the year 2050 [1]. This prediction is increased further in emerging economies like India and China with a manufacturing cement of about half of the worldwide production in 2019 [1,2]. Ordinary Portland cement (OPC) production impacts the environment in various ways. Demolishing limestone reserves, an influential base material of cement results in water and land pollution that disturbs the local ecosystem and animals and plants [3]. The OPC production releases the same carbon dioxide (CO_2_) into the atmosphere, resulting in air pollution [4]. However, some actions isolate CO_2_; it continues to pose damage to the environment [5]. The liberation of CO_2_ due to cement production is responsible for about 7% of the total emission of greenhouse gases and is accountable for about 4% of rising global temperatures [6]. At the same time, there are many issues connected to industrial by-products disposal of ground granulated blast furnace slag (GGBS) and fly ash. They could therefore not be disposed of in land and water caused pollution in land and water. This guided the push to attain a new construction material utilizing these industrial by-products called geopolymer concrete (GPC). Utilizing geopolymer concrete can reduce the impact of cement production on global warming by about 80% [7]. The cement concrete is replaced by geopolymer concrete, which can alleviate the issues resulting from the mining of limestones and disposal of GGBS and fly ash [8]. Duxson et al. [9] reported high durability and early strength exhibited in fly ash and GGBS based geopolymer concrete, respectively [9].

Several studies reported the advantages of GPC as excellent compressive strength, resistance to fire and low creep. Due to the brittle nature of the material, its tensile strength is significantly low [10,11]. A classical technique to enhance the concrete’s post-peak performance is to incorporate fibers as necessary reinforcement. Fibers are very useful in enhancing the mechanical properties, integrity of structures, impact resistance and ductility [12]. Out of various obtainable fibers, steel and glass have high modulus that enhances concrete strength [13,14]. Consequently, several investigations have examined the mechanical properties of fiber-based GPC [15,16,17,18]. However, there is a vast knowledge gap left in the literature on the fracture toughness of fiber-based GPC, mainly with fiber hybridization. Recent findings examined the mode II fracture toughness of concrete with mono fibers and without fibers [19,20]. In contrast, the concrete with three fiber hybridization has been unexamined by researchers, particularly in GPC. Seitl et al. [21] examined the fracture behavior of concrete under mixed-mode using a Brazilian disc test with 40 and 60 mm notch lengths. The inclination angle is used during the testing were 0, 5, 10, 15, 20 and 27.2 degree. The results revealed that the fracture toughness values were 0.903 MPam^1/2^ and 0.903 MPam^1/2^ for the ratio of notch length and radius of the specimen (α/R) were 0.267 and 0.4, respectively. Increasing the inclination angle from 5 to 27.7 degrees with respect to the load applied direction, increasing the fracture toughness from 0.34 to 1.31 MPam^1/2^ for the α/R is 0.267 and from 0.97 to 0.49 for the α/R is 0.4.

Pirmohammad et al. [22] studied the mode I, II and mixed-mode fracture toughness of carbon and kenaf-based asphalt concrete. Three different fiber lengths (4, 8 and 12 mm) and fiber dosage (0.1%, 0.2% and 0.3%) by weight of mixture were used to produce fibrous asphalt concrete. The fracture toughness was evaluated using the semi-circular bend test under −15 °C temperature. The findings indicated that the addition of kenaf and carbon fibers exhibited considerable enhancement in fracture toughness than non-fibrous asphalt concrete. The highest fracture toughness value of fibrous asphalt concrete is attained under pure mode I. Moreover, the best contribution came from 4 mm in length and 0.3% dosage of carbon fiber. Razmi and Mirsayar [23] investigated the mixed-mode I/II fracture toughness of concrete with jute fiber’s addition using a semi-circular bend specimen. Three dosages of jute fibers of 0.1, 0.3 and 0.5% were used with a length of 20 mm. The fracture toughness of plain and fibrous concrete is minimal against pure mode II loading, which indicates less fracture resistance and this is well aligned with earlier findings. As the fiber content increased, the fracture resistance under the mixed mode is increased. Adding a 0.3% dosage of jute fiber in concrete does not significantly improve the resistance to fracture. Aliha et al. [24] reported that the initiation of fractures and stages of propagation are affected significantly by the content of synthetic forta-ferro (SFF) fibers. Fracture toughness was higher in SFF (0.3%) based concrete than plain cement concrete due to the cracking resistance against both tear and tensile loads. Mal et al. [25] investigated the Mode II fracture toughness of hybrid fibrous composites using four test methods viz., double-edge notched prism, double notched cube, Brazilian notched disc, four-point shear and double edge notched prism. Four patterns of hybridization using three fibers such as polypropylene, glass and steel with a cumulative 1.5% dosage were maintained. The ratio of the depth of notch to the specimen’s width (d/w) (0.3, 0.4 and 0.5) was implemented. Findings indicated that the concretes fracture toughness was lessened with an increasing (d/w) ratio for all hybridization patterns. The higher fracture toughness under mode II was achieved from the hybridization of steel, polypropylene and glass fiber. The percentage increases vary from 25% to 30% for the four-point shear and double edge notched prism, from 17% to 18% for the Brazilian notched disc, from 20% to 30% for the double notched cube and from 11.5% to 16% for double-edge notched prism.

A literature review indicates that there are very limited studies on the fracture toughness under mode II with plain concrete and fibrous concrete by mono or hybrid fiber types. The researchers employ several fracture test specimens to evaluate the mode II fracture toughness of various concrete in the past. However, the evaluation of mode II fracture toughness of fibrous geopolymer using the Brazilian notched disc and the double notched cube was not found in any literature and needed special attention. This research aims to investigate the mode II fracture toughness of geopolymer concrete comprising steel, glass, and polypropylene fiber with the cumulative dosage of 1.6% to fill this research gap. The Brazilian notched disc and double notched cube test were employed to evaluate fracture toughness by changing the notch depth to 45, 60 and 75 mm. Since the influence of fiber hybridization on the mode II fracture toughness of the geopolymer concrete is not yet investigated, the research outcomes provided in this research can support the investigators to clarify some doubts on fracture toughness of fibrous geopolymer concrete under mode II conditions.

## 2. Experiential Study

### 2.1. Raw Materials

The main base materials for making fibrous geopolymer concrete were GGBS, fine and coarse aggregate, alkaline activators and fibers.

**Figure 1:**
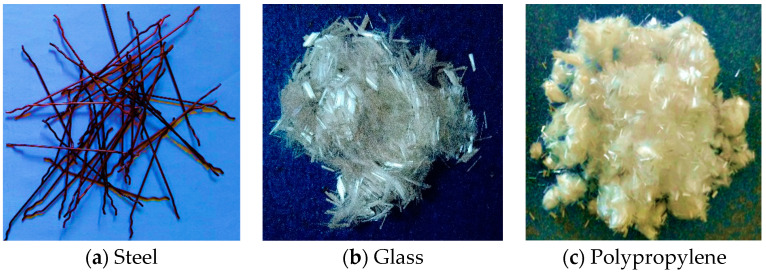
Appearance of fibers used in this study [27].

- GGBS is procured from Astrra chemicals, Chennai, India and its chemical composition were (%): CaO—36.77, SiO_2_—30.97, Al_2_O_3_—17.41, MgO—9.01, SO_3_—1.82, Fe_2_O_3_—1.03, Na_2_O—0.69, K_2_O—0.46. The specific gravity and fineness were 2.9 and >350 m^2^/kg, respectively.
- The fine aggregate used was natural river sand obtained near Thanjavur, India with the specific gravity of 2.65 and fineness modulus of 2.41, in accordance with IS: 383-2016 [26].
- A 12.5 mm crushed granite gravel was used as coarse aggregate as per IS: 383-2016 [26]. Coarse aggregate obtained locally in Thanjavur, India which had a 0.56% water absorption, 2.69 specific gravity, and 1700 kg/m^3^ apparent bulk density.
- The sodium silicate (Na_2_SiO_3_) and sodium hydroxide (NaOH) liquid were used to prepare a 12-molarity solution and these materials were procured from Astrra chemicals, Chennai, India. The flakes of NaOH were dissolved in distilled water to evade the effect of unidentified contaminate in the mixing water. The mix of NaOH was prepared 24 h before casting.
- A new 5D hooked end steel fiber (SF), glass fiber (GF) and polypropylene fibers (PF) were utilized and their properties are shown in Table 1. The appearance of three used fibers is shown in Figure 1. The main reason for adding micro polypropylene and glass fibers is to control the micro cracks effectively. Long steel fibers usually exhibit good bonding between the matrix and fiber and this becomes the controlling criterion for arresting macro-cracks. The hybrid combination of these fibers leads to interconnected micro-cracks and macro cracks in the fracture region, resulting in an effective crack control during the crack proliferations.

### 2.2. Specimen Preparation

The mixing composition and ingredients used for producing geopolymer are demonstrated in Table 2. Eight mixtures were prepared with steel, glass and polypropylene fibers. The first mixture was kept as a reference concrete is denoted by reference concrete (RC). The next three mixtures were mono-reinforced with polypropylene, glass and steel fiber with a dosage of 0.3, 0.3 and 1.6, respectively. The next four mixtures were prepared with hybridization of two fibers, and the final mixture was with the hybridization of three fibers. The dosage of fiber in hybridization is limited to 1.6% due to the non-uniform fiber distribution that has tendency towards conglobate which leads flaws in composite and weakening the interfacial transition zones that causes strength loss. The dosage of fiber was chosen based on the concrete’s total volume. The GGBS of 100% was used as the source of aluminosilicate that greatly reduces the setting time than fly ash. All casted specimens were kept in open air for 28 days.

### 2.3. Experimental Setup

The mode II fracture tests were conducted using two methods, viz., double-notch cubic (DNC) specimens and Brazilian center notched disc (BCND) specimen in conformity with a concept presented in [25]. The mode II fracture was evaluated from DNC test by considering three notch depth to cube width ratio (0.3, 0.4 and 0.5) and BCND test by considering three notch length to disc diameter ratio (0.3, 0.4 and 0.5) were used in conformity with [25]. The geometry of the specimens used for Mode II fracture is illustrated in Table 3. All the specimens were tested in 300 T capacity compression testing machine.

## 3. Results and Discussion

### 3.1. Influence of Fiber Type and Fiber Hybridization

**Figure 2:**
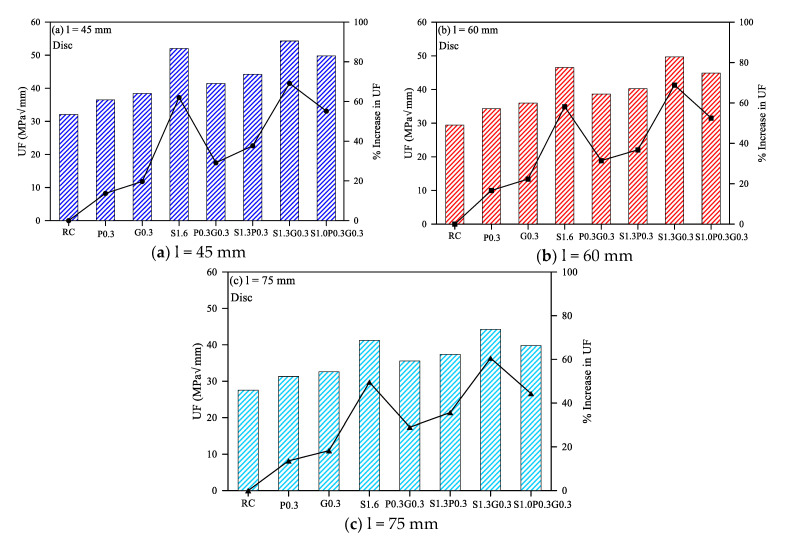
Mode II disc specimens’ fracture energy of the eight geopolymer mixtures.

The fracture toughness results of the disc specimens made of the eight geopolymer mixtures are presented in Figure 2a–c for the different adopted notch depths. The average of the three fracture toughness value is provided in Table 4, and the maximum and minimum difference between these values were less than 2%. It is obvious in the three figures that the mono-fibrous mixtures that were reinforced with only one fiber type were listed first, preceded by that of the reference non-fibrous mixture and followed by the hybrid fiber-reinforced mixtures. It is obvious that the fracture toughness (UF) for all mixtures ranged from approximately 27.5 to 54.3 MPa√mm. Comparing the three mono-fibrous mixtures, it is obvious in Figure 2a that the discs reinforced with 1.6% of steel fiber (S1.6) exhibited noticeably higher UF compared to the two synthetic fiber-reinforced mixtures, while the one with glass fiber (G0.3) showed slightly better performance compared to the polypropylene fiber-reinforced mixture (P0.3). Similar result sequences were noticed for the three mixtures in Figure 2b,c for the deeper notch depths (60 and 75 mm). Considering the lowest UF of the three mixtures (P0.3) as a datum, the percentage enhancements in UF due to changing the fiber type to glass fiber and steel fiber were in the ranges of 4.1% to 5.2% and 31.6% to 42.5%, respectively, for the three notch depths, which reflect the slightly better performance of glass fiber and significant improvement of steel fiber. This trend can be attributed to the superior physical properties of higher tensile strength and modulus of elasticity of steel fibers compared to the used synthetic fibers, where the modulus of elasticity plays a vital role in the total fracture toughness of materials. Similarly, the modulus of elasticity of glass fiber is much higher than that of polypropylene fiber, which enhanced the retained fracture toughness. A higher modulus of elasticity and tensile strength results in a stiffer matrix, which delays the first crack initiation and increases the ultimate strength [25,27,29].

**Figure 3:**
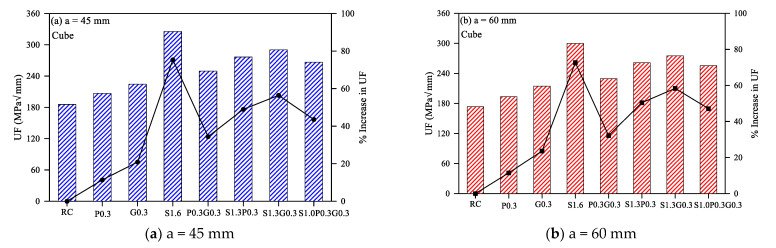

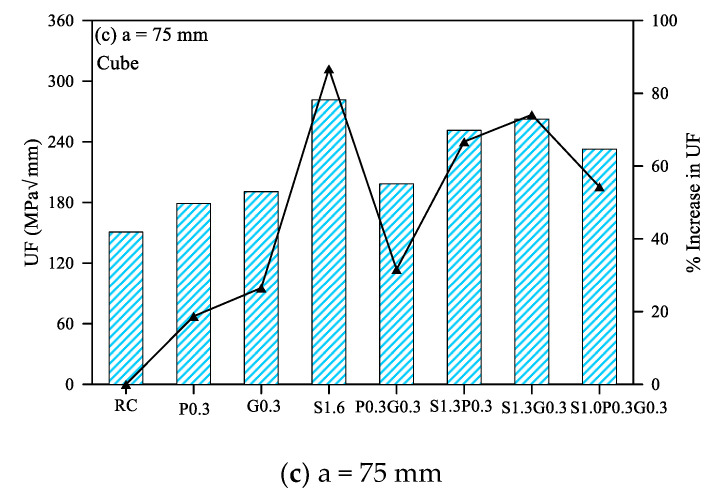
Mode II cube specimens’ fracture energy of the eight geopolymer mixtures.

A similar trend of results is also noticed for the mode II fracture cube specimens. The cubes also showed distinguishably higher UF for S1.6 mixture compared to P0.3 and G0.3 mixtures. This behavior is clear for the three notch depths in Figure 3a–c. Similarly, the inclusion of glass fiber (G0.3) led to higher UF compared to the use of polypropylene fibers (P0.3). The UF of G0.3 was higher than that of P0.3 by 6.6% to 10.9%, while S1.6 was higher by 54.9% to 57.4% compared to P0.3 for the three notch depths. This result trend is also attributed to the higher stiffness and ultimate failure load of steel fibers compared to synthetic fibers and of glass fiber compared to polypropylene fiber. Another possible cause of this trend is the higher length of steel fibers. Short fibers have the potential to arrest the micro-cracks within the binder matrix, while longer fibers are capable to arrest the propagation of macro cracks in the concrete structure [29,30,31,32,33]. For this reason, longer fibers are believed to be more efficient in enhancing the total fracture toughness of the adopted mixtures than shorter ones. It should be reminded here that the length of the used fiber is 60 mm, while it is only 13 and 15 mm for the glass and polypropylene fibers. The volume fractions used can also be considered a crucial factor that impacts the resulted strength and toughness of the matrix. For the mono-fiber reinforced mixtures, the steel fiber volume fraction was 1.6%, while those of glass and polypropylene fibers were 0.3% only. However, it should be kept in mind that the specific weight of steel is also much higher than the other two synthetic fibers, which reduces by weight fraction and total number of used fibers compared to the synthetic fibers.

The following four bar charts in the six figures shown in Figure 2 and Figure 3 compare the mode II fracture toughness of the hybrid-fibrous mixtures. The hybrid fibrous mixtures followed a similar fashion of improvements in UF to that of mono-fibrous specimens. It is obvious that P0.3G0.3 mixture exhibited the lowest UF values among the four hybrid mixtures for both disc and cube specimens and for all notch depths. This is attributed to the addition of steel fibers in the other mixtures, which better improved their stiffness, ultimate strength and energy absorbance capacity. The synergic effect of steel fiber with synthetic fiber led to higher fracture energies due to two hybrid actions. The first is the physical synergy, which benefits of the stiffer, higher strength and tougher steel fiber and the more flexible polypropylene or glass fiber, which assures stiffer matrix with higher load capacity and higher plastic toughness. On the other hand, the geometrical synergy is accomplished by the use of synthetic micro-fibers that delay the initiation of the cracking and steel macro-fiber that arrests the propagation of macro cracks within the post-cracking stage [28]. As shown in Figure 2 and Figure 3, the performance of the glass fiber was also better than that of the polypropylene fiber for a fixed steel fiber fraction (S1.3P0.3 and S1.3G0.3), which follows a similar trend to that discussed for mono-fibrous mixtures.

Comparing the eight mixtures, it can be summarized that the mono-fibrous mixture with pure 1.6% of steel fiber (S1.6) and the hybrid fibrous one with steel and glass fibers (S1.3G0.3) retained the highest fracture toughness values. To specify, S1.3G0.3 was the mixture with the highest disc UF values as shown in Figure 2, while S1.6 exhibited the highest cube UF values as shown in Figure 3. However, their values were in general comparable with percentage differences between the two mixtures, for both disc and cube specimens and for all notch depths, ranged from 4.4% to 10.8%. It is also obvious in the figures that the UF values of the seven fibrous geopolymer mixtures were higher than that of the non-fibrous reference geopolymer mixture. The black lines in the six figures of Figure 2 and Figure 3 show the percentage increase in UF in each of the seven fibrous mixtures over the reference mixtures. It is shown that the percentage increase in disc UF of fibrous specimens was in the range 13.6% to 69.1%, while it is in the range of 11.4% to 86.7% for cube specimens. This is of course attributed to the potential of all fiber types to arrest the cracking leading to tougher material with higher energy absorbance capacity.

Previous studies that used steel fiber, polypropylene fiber and other synthetic fibers revealed a distinguished superiority of steel fibers, both in mono-fashion or when combined with synthetic fibers, where it could better improve the stiffness, tensile strength, flexural strength, ductility, shear strength and energy absorbance capacity [34,35,36,37,38,39,40,41,42,43], which totally supports the obtained results in this research. Scorza et al. [44] tested different combinations of different length steel and polypropylene fibers and indicated that steel mono-fiber reinforced mixture retained the best fracture results and post-peak performance under mode I fracture test. On the other hand, Shao and Wang [45] showed that the mode I fracture toughness of hybrid steel-polypropylene mixtures, with various percentage combinations, was higher than the steel fiber-reinforced mixture by only 0.4 to 2.7%, while for the same volumetric content, the steel fiber-reinforced mixture retained 26.5% higher than the mono-polypropylene mixture, which support that the main role in improving the mixture fracture properties is related to the incorporation of steel fibers.

The fibers distribution in geopolymer composites were not even, and they oriented randomly due to the influence of several parameters (e.g., properties of steel fiber, placing methods, boundary forms and vibration) during the process of fabrication. This makes ideal structural members of geopolymer composites under external forces in more than two directions (e.g., shell roofs and elevated slabs). A uniform fiber distribution in the direction of tensile stress distribution is vital for structural components under the one direction of external forces (e.g., beams). The non-uniform and randomly oriented fiber distribution prevents the widespread use of fibrous geopolymer composites structures. However, a non-uniform distribution is widespread and even unavoidable, particularly for flexible synthetic fibers.

The improving fracture toughness is due to the steel fibers, which displaced a beneficial effect in the tension zone. The cracks were hindered through the good interlocking between them in the macro and micro levels. During the application of the load, the enhancement of composites is achieved by long fibers, which restrains the propagation of macro cracks, and the tensile stresses were transmitted effectively after crack development. Conversely, the short fibers in composites can bridge micro-cracks efficiently and delay their development due to good bonding between the fibers and matrix.

### 3.2. Influence of Notch Depth 

**Figure 4:**
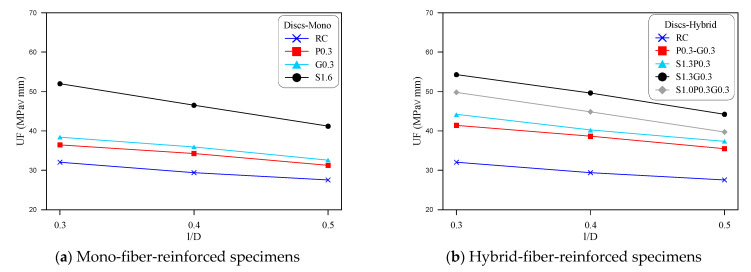
Mode II disc specimens’ fracture energy for different notch depth-specimen diameter (l/D) ratios.

**Figure 5:**
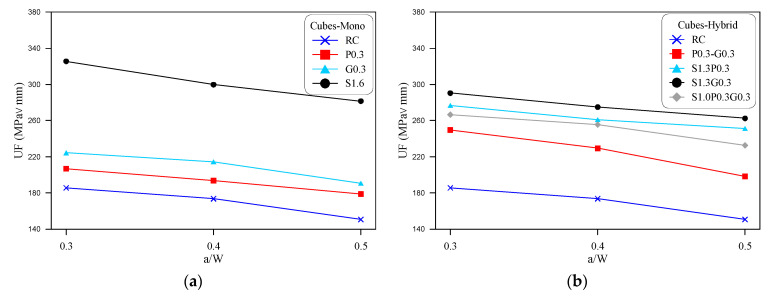
Mode II cube specimens’ fracture energy for different notch depth-specimen width (a/W) ratios.

Comparison of the retained fracture toughness values of the eight mixtures are shown in terms of the notch depth to the diameter of discs in Figure 4 and notch depth to the cube depth (width) in Figure 5. Figure 4a and Figure 5a illustrate the comparisons for the mono-fibrous mixtures, while Figure 4b and Figure 5b show the comparisons for the hybrid fibrous mixtures. As described earlier, three notch depths of 45, 60 and 75 mm were considered as one of the study parameters for both types of specimens. This notch depth when divided by the specimen’s depth (diameter or width) gives the ratios of 0.3, 0.4 and 0.5 as shown in the horizontal axes of Figure 4 and Figure 5. The adopted notch depth/specimen depth ratios were selected based on the recommendations of previous studies [25,45].

Figure 4 shows implicitly that, regardless of the mixture type, the fracture toughness decreases almost linearly with the increase of notch depth which is presented as the ratio of notch depth to disc diameter (l/D). As concluded from the previous section, S1.6 and S1.3G0.3 exhibited the highest UF values among all mixtures, which is also obvious in Figure 4 and Figure 5. The decrease of the retained toughness with notch depth increase is self-explained, where a deeper notch means shorter fracture path, which accelerates the failure of the specimen under lower loads. Hence, as the specimen depth was fixed as 150 mm, increasing the notch depth from 45 to 60 and 75 mm means that the rest concrete depth, that is supposed to withstand the applied loads, was reduced from 105 to 90 mm and then to 75 mm. Similar trend of results was also obtained by previous researches using four different mode II fracture tests [25,46].

### 3.3. Influence of Test Type 

It is obvious by comparing Figure 2 and Figure 3 that cube specimens retained much higher fracture toughness values than the disc specimens. For all mixtures, the UF of cubes were approximately 5.4 to 6.8 times those of the disc specimens. However, it was introduced that the depth (diameter of the disc and side length of the cube) of both types of specimens was 150 mm, while they were cast with different thicknesses of only 60 mm for the disc and 150 mm for the cube specimens. Therefore, for better and fairer comparison, the UF vales of both specimens were normalized to their thicknesses. 

**Figure 6:**
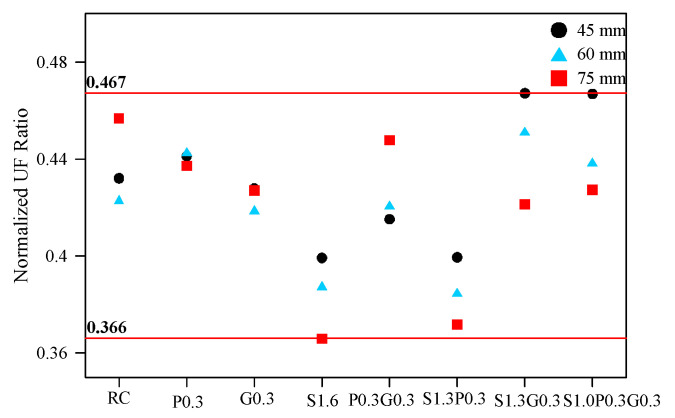
Ratio of normalized discs fracture energy to normalized cubes fracture energy for the eight mixtures.

**Figure 7:**
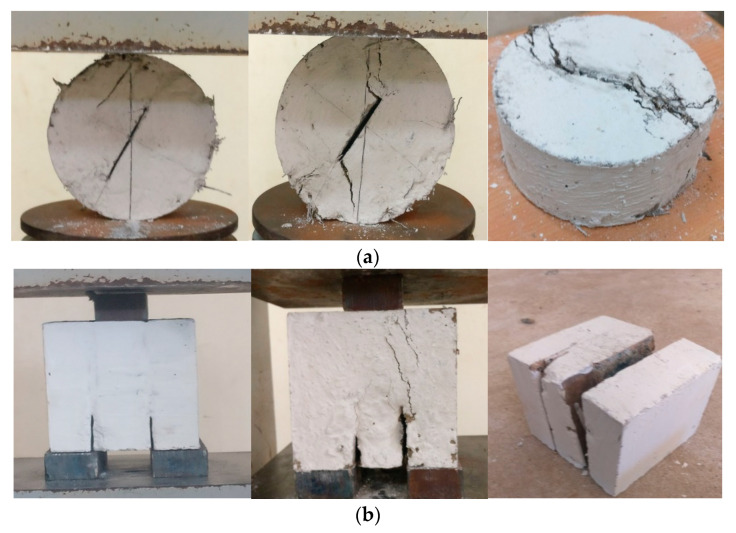
Failure pattern of specimens: (**a**) Brazilian cylindrical specimens and (**b**) Double notched cube.

Comparisons were made between the normalized UF values of disc and cube specimens in Figure 6, which shows the ratio of disc normalized UF to cube normalized UF results. It is obvious in the figure that the fracture toughness of the cubes is still more than twice that of discs, where the ratio was in the range of approximately 0.37 to 0.47. This yields to a firm conclusion that double notched cube specimens exhibited more than twice the fracture toughness of Brazilian notched disc specimens. This result can be attributed to two reasons, which are both related to the configuration of the specimens. The first is that in the disc specimens the stresses are concentrated along one path, which is the notch line. Hence, the failure of the specimen would be along the specified path with possible inclinations related to the angle of load application. This behavior is clear in the cracking and fracture of the disc specimens shown in Figure 7a, where the crack was initiated along the single planned path which means that stresses were concentrated along this path leading to a more systematic failure. On the other hand, the two notches of cube specimens would help distribute the applied load and hence distribute the induced stresses along more than one path. This behavior is also clear in the cracking and failure patterns of cube specimens as shown in Figure 7b. As a result, the retained failure loads and hence the fracture toughness would definitely be higher than disc specimens. The second reason is that the better planned cracking and failure path of discs would result in a combined mode I and mode II fracture, which may lead to the tensile failure of the discs before being failed in shear [47]. The research outcomes obtained from this study is to deliver a baseline information for continuing a deeper research work on the fracture toughness of concrete [48,49,50,51,52,53].

## 4. Conclusions

Two mode II fracture tests were conducted in this research on seven mono- and hybrid-fiber reinforced geopolymer concrete mixtures in addition to a plain reference mixture. Brazilian center notched discs and double notched cubes were tested for the eight mixtures. The test results obtained from the experimental research yielded the following conclusions:

1. All fibrous specimens retained noticeably higher fracture toughness compared to plain specimens. A percentage development in the fracture toughness, compared to the reference mixture, of 13.6% to 69.1% was obtained for disc fibrous specimens, while that of fibrous cube specimens was higher than the reference specimens by 11.4% to 86.7%. This performance is attributed to the fibers crack bridging capability, which results in higher cracking and ultimate loads and more ductile behavior leading to tougher behavior with higher absorbed energy.
2. The steel mono-fibrous mixture (S1.6) and the hybrid fibrous one with steel and glass fibers (S1.3G0.3) were the mixtures with the highest fracture toughness values in both tests. However, the percentage differences between the two mixtures were limited to 4.4% to 10.8%, which reflects the crucial and superior effect of the used steel fibers compared to the synthetic fibers. This result is attributed to the longer steel fiber and its higher strength and modulus of elasticity compared to synthetic fibers.
3. Regardless of the mixture type and test type, the fracture toughness exhibited an approximately linear decrease with the increase of notch depth. For instance, the fracture toughness values of the discs made from the mixture S1.6 were recorded to be 52.0, 46.5 and 42.2 MPa√mm for notch depth/diameter ratios of 0.3, 0.4 and 0.5, respectively. This trend of decrease is attributed to the increases in notch length, where deeper notches mean shorter fracture paths, which accelerate the failure of the tested specimens.
4. Double notched cube specimens retained apparently higher fracture toughness compared to the Brazilian notched disc specimens. The ratio of normalized fracture toughness of the disc specimens to that of their corresponding cube specimens ranged from 0.37 to 0.47. This result can be attributed to the concentration of stresses along one defined path in the disc specimens compared to the multi-path stresses in cube specimens. In addition, the accompanied tensile stresses in disc specimens may lead to mode I fracture before the designed mode II fracture.

## Figures and Tables

**Table 1:** Properties of fiber.

Properties	PF	GF	SF
Tensile Strength (MPa)	360	1400	1050
Density (kg/m^3^)	910	2600	7850
Diameter (mm)	0.095	1	0.9
Length (mm)	13	15	60

**Table 2:** Mix composition of geopolymer composite [28].

S. No	Mix Id	GGBS (kg/m^3^)	FA (kg/m^3^)	CA (kg/m^3^)	W/B Ratio	NaOH (kg/m^3^)	Na_2_SiO_3_ (kg/m^3^)	SF (%)	PP (%)	GF (%)
1.	RC	414	515	956	0.5	69	138	0	0	0
2.	P0.3							0	0.3	0
3.	G0.3							0	0	0.3
4.	S1.6							1.6	0	0
5.	P0.3G0.3							0	0.3	0.3
6.	S1.3P0.3							1.3	0.3	0
7.	S1.3G0.3							1.3	0	0.3
8.	S1.3P0.3G0.3							1	0.3	0.3

CA: coarse aggregate, FA: fine aggregate, SF: steel fiber, PP: polypropylene fiber, GF: glass fiber.

**Table 3:** Mode II fracture toughness test geometries.

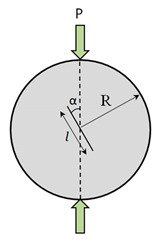 Brazilian center notched disc	K_IIe_ = −$\frac{2P}{t}$ $\sqrt{\frac{\beta }{\pi R}}$ $\left\{\begin{array}{c} A_{0}-\beta^{2\;}\left(A_{0}+\frac{1}{2}\;A_{2}\right)+\beta^{4}\left(-\frac{1}{8}A_{0}+\frac{1}{4}A_{2}+\frac{3}{8}A_{4}\right)\\ +\beta^{6}\left(A_{0}-\frac{1}{16}A_{2}+\frac{1}{8}A_{4}+\frac{5}{16}A_{6}\right)\\ +\beta^{8}\left(-\frac{17}{64}A_{0}+\frac{3}{8}A_{2}-\frac{5}{128}A_{4}+\frac{5}{64}A_{6}+\frac{35}{128}A_{8}\right) \end{array}\right\}$
	t = 60 mm, R = 75 mm, $\beta =\frac{l}{2R}$
	α = Notch inclination angle = 30°
	A_0_ = sin 2α, A_2_ = 2(sin 4α − sin 2α), A_4_ = 3(sin 6α − 2sin 4α)
	A_6_ = 4(sin 8α − 3sin 6α), A_8_ = 5(sin 10α − 4sin 8α)
	l = 45, 60, 75 mm
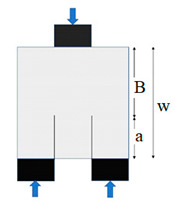 Double notched cube test	K*_IIC_* = $\frac{5.11}{2Bw}$ P_Q_ (πl)^1/2^
	Cube 150 mm
	l or a = 45, 60, 75
	w = 150
	B = w − l

**Table 4:** Fracture toughness test results.

Mix Id	Double Notched Cube Test			Brazilian Center Notched Disc		
	l = 45 mm	l = 60 mm	l = 75 mm	l = 45 mm	l = 60 mm	l = 75 mm
RC	185.70	173.66	150.77	32.10	29.41	27.56
P0.3	206.81	193.58	179.00	36.50	34.31	31.31
G0.3	224.50	214.63	190.75	38.42	35.98	32.59
S1.6	325.60	299.83	281.54	52.00	46.52	41.21
P0.3G0.3	249.56	229.51	198.38	41.45	38.66	35.54
S1.3P0.3	276.62	261.23	251.36	44.20	40.23	37.38
S1.3G0.3	290.46	275.00	262.51	54.29	49.66	44.25
S1.3P0.3G0.3	266.59	255.49	232.68	49.80	44.86	39.78

## Data Availability

Data sharing not applicable.
